# Functional alterations due to post-COVID-19 lung lesions — lessons from a multicenter V/Q SPECT/CT based registry

**DOI:** 10.1007/s00259-026-07828-z

**Published:** 2026-03-17

**Authors:** Pierre-Benoit Bonnefoy, Pierre Pascal, Quentin Ceyrat, Samuel Burg, Micheline Razzouk-Cadet, Caroline Moreau Triby, Ingrid Biancheri Mounicq, Jean-Cyril Bourre, Pierre-Yves Salaun, Pierre-Yves Le Roux

**Affiliations:** 1 Service de Médecine nucléaire, CHU de Saint-Etienne Hôpital Nord – Avenue Albert Raymond, Saint-Etienne, 42055 France; 2 Service de Médecine nucléaire, CHU Toulouse Rangueil, Toulouse, France; 3 Service de Médecine nucléaire, Centre d’imagerie Fonctionnelle, Bordeaux, France; 4 Service de Médecine nucléaire, GCS Médecine nucléaire, Ajaccio, France; 5 Service de Médecine nucléaire, CHU de Nice, Nice, France; 6 Service de Médecine nucléaire, CHU de Lyon, Lyon, France; 7 Service de Médecine nucléaire, CH Angoulême, Angoulême, France; 8 Service de Médecine nucléaire, CH Chambéry, Chambéry, France; 9 Univ Brest, CHU Brest, Médecine Nucléaire, GETBO, UMR1304, Brest, France

**Keywords:** V/Q SPECT/CT, Long COVID, Pulmonary Embolism, SARS-Cov-2

## Abstract

**Purpose:**

The COVID-19 pandemic has left many survivors with persistent respiratory symptoms such as dyspnea, chronic cough, and reduced exercise capacity. Fibrosis-like abnormalities on high-resolution CT are frequently reported in theses patient. This study aimed to describe the morphological pulmonary abnormalities identified on V/Q SPECT/CT after COVID-19 infection and to evaluate their functional consequences on regional ventilation and perfusion. We also sought to identify clinical predictors of abnormal V/Q SPECT/CT findings.

**Methods:**

This retrospective multicenter study was based on a national registry of patients referred for V/Q SPECT/CT because of persistent respiratory symptoms after COVID-19 infection. CT abnormalities were characterized using standard radiological criteria. Ventilation and perfusion were visually graded using a five-point scale. Clinical, demographic, and acute COVID-19 characteristics were analyzed to identify predictors of abnormal imaging.

**Results:**

Among the 217 included patients (mean age 58 ± 15 years), 122 (56%) presented pulmonary abnormalities on V/Q SPECT or CT. The most frequent parenchymal abnormalities were reticulations, consolidations, atelectasis, emphysema, and ground-glass opacities. Emphysema, consolidations, and fibrosis were associated with the most severe impairments of both ventilation and perfusion. Age and pre-existing chronic lung disease were the strongest predictors of abnormal imaging, while a mild acute COVID-19 course without oxygen therapy was protective.

**Conclusion:**

V/Q SPECT/CT provides a comprehensive structural and functional assessment of long COVID pulmonary sequelae. It allows the differentiation between reversible inflammatory lesions and persistent structural abnormalities with lasting functional consequences.

## Introduction

The COVID-19 pandemic has left a substantial proportion of survivors facing persistent symptoms, transforming what was initially an acute viral illness into a chronic condition now referred to as post-COVID syndrome or long COVID [1, 2]. Respiratory manifestations are among the most frequently reported symptoms, including dyspnea, chronic cough, chest discomfort, and reduced exercise capacity, affecting up to one-third of individuals several months after the acute infection [3]. These persistent symptoms have a major impact on quality of life and daily functional abilities.

Long COVID represents a heterogeneous clinical entity, and the mechanisms underlying persistent respiratory symptoms remain partially understood. Several pathophysiological pathways have been proposed, including residual parenchymal damage, pulmonary microvascular and coagulation abnormalities, autonomic dysfunction, and functional or psychogenic disorders [4–6]. Current guidelines emphasize the importance of identifying organic causes that could be addressed with standard, pathology-specific treatments [7–10]. Moreover, many patients continue to experience unexplained respiratory symptoms, underscoring a frequent dissociation between symptoms and conventional morphological imaging than remain normal in a half of patient [11]. In this selected population, rehabilitation may contribute to symptom improvement [12]. Nevertheless, despite such approaches, chest computed tomography (CT) findings do not influence the decision of pneumologists regarding the diagnosis and management of pulmonary long coronavirus disease [13]. This frequent mismatch emphasizes the need for reliable diagnostic tools capable of detecting subtle functional impairments and distinguishing reversible inflammatory abnormalities from fixed structural sequelae.

Ventilation/perfusion (V/Q) single photon emission computed tomography/computed tomography (SPECT/CT) imaging may help bridge this diagnostic gap. Although, V/Q SPECT/CT has been only marginally explored in the context of COVID, with most nuclear medicine studies focusing either on the acute phase [14, 15] or on thromboembolic complications [11]. Owing to its ability to detect pulmonary vascular involvement [16], lung SPECT is currently proposed by the European Society of Cardiology and European Respiratory Society (ESC/ERS) guidelines for the evaluation of long COVID [10] particularly to exclude vascular pulmonary obstruction in persistent respiratory symptoms after COVID-19, even in the absence of documented pulmonary embolism during the acute phase. In a previous work based on a national registry including patients who underwent lung scintigraphy for post-COVID respiratory impairment, we reported that the rate of post-embolic conditions was actually low in this setting (2.6%). However, only 45% had normal lung CT while approximately 55% of patients showed abnormalities on V/Q SPECT/CT [11]. These findings underscored the heterogeneity and complexity of post-COVID respiratory presentations. Beyond the exclusion of thromboembolic disease, V/Q SPECT/CT offers simultaneous structural and functional evaluation of the lungs within a single examination [17–19], allowing the detection of subtle parenchymal abnormalities and their consequences on regional ventilation and perfusion.

The primary objective of the present study was to characterize the morphological abnormalities identified on V/Q SPECT/CT performed for persistent respiratory symptoms after COVID-19 and to assess their functional consequences on regional ventilation and perfusion. A secondary objective was to identify clinical predictors associated with abnormal V/Q SPECT/CT findings in patients with long COVID.

## Materials and methods

### Study design and population

This retrospective multicenter study was conducted using a national registry established by the French Society of Nuclear Medicine (SFMN) working group on lung scintigraphy. The registry prospectively collected V/Q SPECT/CT examinations performed in patients presenting with persistent respiratory symptoms after SARS-CoV-2 infection. Two nationwide calls for participation were disseminated to all members of the society by email in March and September 2022. Participating center provided anonymized clinical data consistent with demographic information, medical history, details of the acute infection, and symptoms present at the time of imaging and imaging data stored as DICOM files to a secure platform (DOXACA, Nancy, France).

The study protocol was approved by the Nuclear Medicine Research Ethics Committee (CEMEN 2022-03). Each participating center obtained a documented non-opposition form from all patients prior to data transmission.

Patients were eligible if they were aged 18 years or older, had a confirmed history of COVID-19 infection, and were referred for V/Q SPECT/CT because of persistent respiratory symptoms consistent with ongoing symptomatic COVID-19 (4 to 12 weeks) or long COVID (> 12 weeks) [7].

### Imaging and central review protocolI

V/Q SPECT/CT were performed using gamma cameras equipped with low-dose CT capability. Lung perfusion imaging was performed after intravenous injection of 99mTc-labeled macroaggregated albumin. Lung ventilation imaging was performed using either 99mTc-Technegas or 81mKr gas depending on local availability and center practice [20]. Only complete V/Q SPECT/CT acquisitions were included in the centralized review. The review process was retrospective and blinded from clinical information.

Low-dose CT images were systematically reviewed to identify pulmonary abnormalities. When present, the extent of abnormalities was visually estimated using a segmental system in which each pulmonary segment represented approximately 5% of total lung volume [21]. Lesions were also localized according to their predominant topography (subpleural, intermediate, or perihilar). Each abnormality was classified according to radiological semiology, using definitions based on the Fleischner Society terminology [22]. Emphysema corresponded to irregular enlargement of distal airspaces with destruction of alveolar walls. Ground-glass opacities were defined as a hazy increase in attenuation that preserved vascular and bronchial markings. Consolidation referred to homogeneous opacification that obscured these structures. Crazy paving was recognized when ground-glass opacities were associated with thickened interlobular and intralobular septa. Reticulation consisted of a mesh-like network of septal and intralobular lines. Atelectasis indicated regional loss of lung volume due to airspace collapse. Pulmonary fibrosis encompassed reticulation with architectural distortion and traction bronchiectasis, with or without honeycombing. These definitions were kept concise for clarity while preserving essential diagnostic criteria.

Beyond parenchymal characterization, extra-pulmonary findings were also systematically assessed. Pleural and pericardial effusions, mediastinal lymphadenopathy, and abnormalities of the tracheobronchial tree were recorded when present. Cardiomegaly was defined by a heart-to-mediastinum ratio greater than 1. The diameter of the pulmonary artery trunk was measured from the mean of three measurements taken along its course, and a threshold of ≥ 33 mm was used based on published diagnostic performance [23].

**Fig. 1:**
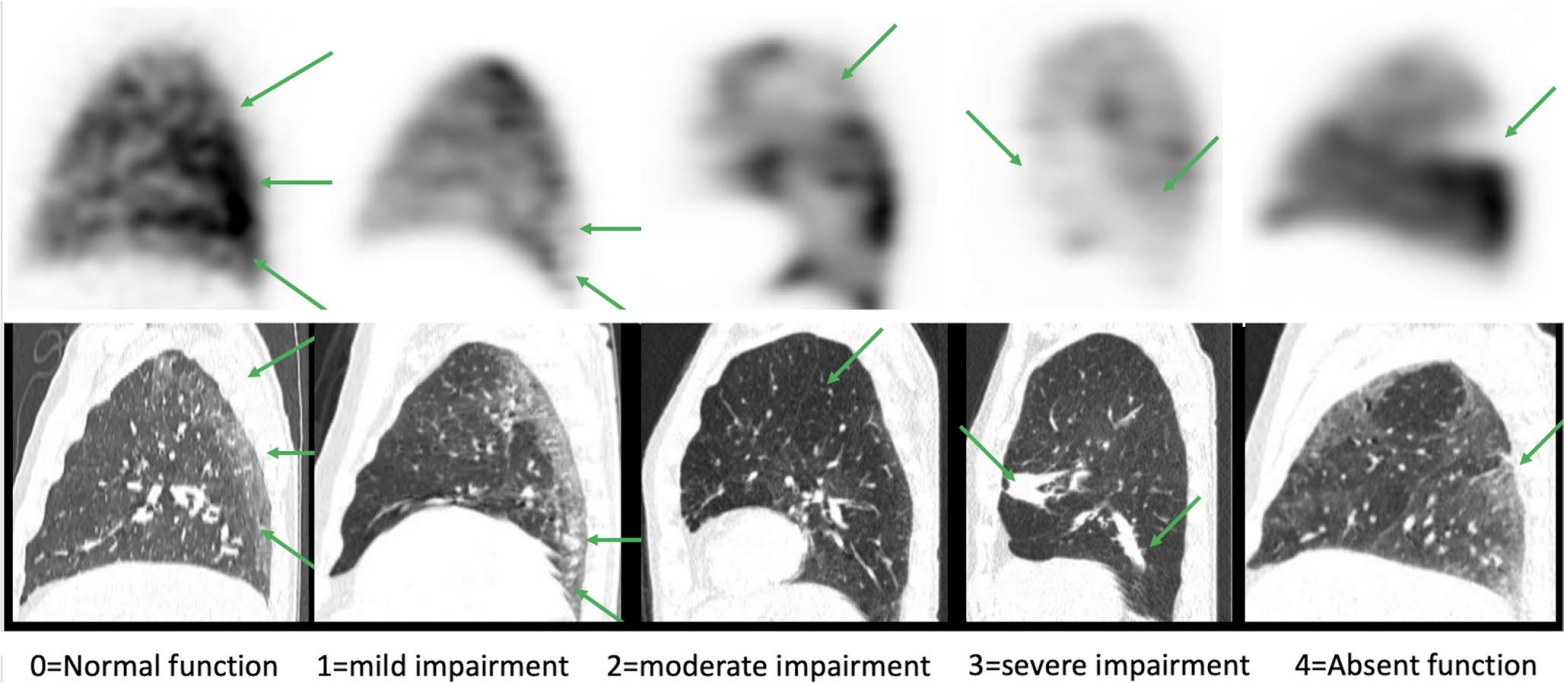
Description of the 5-point scale used for ventilation and perfusion semiquantitative evaluation in a cohort of patient explored for persistent symptom after COVID infection. When CT lesions were visible, perfusion and ventilation were independently evaluated using visual score. Score 0 corresponds to a normal function, 1 to a mild impairment, 2 to a moderate impairment, 3 to a severe impairment, 4 to an absent function. Images are related to pulmonary perfusion scoring related to CT lesions visible (green arrows)

Ventilation and perfusion were analyzed in all patients presenting parenchymal abnormalities involving at least 50% of one lung segment. This threshold was selected to exclude very small lesions considered unlikely to produce clinically relevant functional impairment. When several lesion types were present in the same patient, the most extensive abnormality was analyzed. If a second abnormality involved a similar lung volume, it was evaluated separately. Ventilation and perfusion were independently graded using a 5-points score based on a visual grading scale compared to normal uptake on healthy area. This visual grading scale was adapted from the visual index proposed by Meyer et al. [24] and previously applied for the functional evaluation of acute COVID-19 lung lesions [25]. Ventilation and perfusion were independently graded using a score based on a visual grading scale compared to normal uptake on healthy area (See Fig. 1: 0 = normal function, 1 = mild impairment, 2 = moderate impairment, 3 = severe impairment, 4 = absent function). When present, the extent of abnormalities was visually estimated using a segmental system in which each pulmonary segment represented approximately 5% of total lung volume [21].

### Statistical analysis

Descriptive statistics were used to summarize patient characteristics. Categorical variables were expressed as numbers and percentages. Continuous variables were reported as means ± standard deviations or, as median and range, as appropriate. Comparisons between groups for continuous variables were performed using Welch’s t-test, which was chosen instead of the classical Student’s t-test because it does not assume equality of variances and is more robust in the presence of unequal sample sizes or heteroscedasticity. Comparisons of categorical variables were conducted using the chi-square test when expected cell counts were sufficient; when at least one expected frequency was below 5, the Fisher’s exact test was preferred due to its superior accuracy in small-sample conditions. All analyses and graphical representations were performed using RStudio (Version 1.3.1093).

Univariate analyses were conducted to explore associations between clinical, functional, and imaging variables and the presence of abnormal V/Q SPECT/CT findings. Analyses were performed using GraphPad Prism (Version 10.4.1). Depending on variable type, comparisons were made using chi-square or Fisher’s exact tests for categorical variables and Student’s t-test or Mann–Whitney U tests for continuous variables. Statistical significance was defined as *p* < 0.05.

## Results

### Study Population

A total of 217 patients were included in the registry. The demographic and clinical characteristics of the population have been previously reported in our previous work [11]. These data are summarized in Table 1. The mean age was 58 ± 15.4 years (range 18–94), and men were more numerous than women, with a sex ratio of 1.5. More than half of the patients (*n* = 128, 56.1%) required hospitalization during the acute phase of COVID-19. Among them 111 (86.7%) received oxygen therapy, 71 (55.4%) were admitted to an intensive care unit, and 48 (37.5%) required invasive mechanical ventilation.

**Table 1:** Study population

	General population		Normal V/Q and/or lung CT		Anormal V/Q and/or lung CT		
	217		95		122		*p*
**Demographical data**							
Age (Mean; SD)	*58.1*	(*±* 15.4)	51.5	(*±* 14.31)	*63*,*17*	(*±* 14,36)	
*Age 18–35 (n; %)*	19	(8.8%)	14	(14.7%)	5	(4.1%)	*P* < 0.01
*Age 36–50 (n; %)*	49	(22.6%)	31	(32.6%)	18	(14.8%)	*P* < 0.01
*Age 51–65 (n; %)*	78	(35.9%)	36	(37.9%)	42	(34.4%)	NS
Age > 65 (n; %)	70	(32.3%)	13	(13.7%)	57	(46.7%)	*P* < 0.001
NA (n; %)	1	(0.5%)			1	(0.8%)	
Gender Male (n; %)	130	(59.9%)	46	(48.4%)	84	(68.9%)	
**Background**n							
Chronic lung disease							
*None/unknown (n; %)*	187	(86.2%)	88	(92.6%)	99	(81.1%)	*P* = 0.32
*Chronic lung disease (n; %)*	27		5	(5.3%)	22	(18.1%)	*P* < 0.01
COPD (n; %)	13	(6%)	3	(4.2%)	10	(7.4%)	
Emphysema (n; %)	6	(2.8%)	0	(1.1%)	6	(3.3%)	
Fibrosis (n; %)	1	(0.5%)	0	(0.0%)	1	(0.0%)	
Active cancer (n; %)	0	(0%)	0	(0.0%)	0	(0.0%)	
Other lung disease (n; %)	10	(4.6%)	2	(0.0%)	8	(0.0%)	
Data not available	3	(1.4%)	2	(2.1%)	1	(0.8%)	
Chronic heart disease (n; %)	24	(11.1%)	6	(6.3%)	18	(14.8%)	*P* = 0.053
Renal failure (n; %)	7	(3.2%)	1	(1.1%)	6	(4.9%)	NS
Prior pulmonary embolism (n; %)	12	(5.5%)	5	(5.3%)	7	(5.7%)	NS
**Post COVID lung scan**							
Ventilation tracer (n; %)							
*99mTc-Technegas (n; %)*	121	(55.8%)	65	*(68.4%)*	56	*(45.9%)*	*P* < 0.001
81m-Krypton (n; %)	96	(44.2%)	30	*(31.6%)*	66	*(54.1%)*	*P* < 0.001
Time from infection to examination (days) (Mean; SD)	*187.2*	*(± 140.1)*	*193.56*	*(± 145.1)*	*182.16*	*(± 136.48)*	
*Ongoing symptomatic COVID-19 (n; %*)	*16*	*(7.4%)*	10	*(10.5%)*	6	*(4.9%)*	NS
*Long covid (n; %)*	*201*	*(92.6)*	85	*(89.5%)*	116	*(95.1%)*	NS
Symptoms at time of scan							
*Dyspnea (n; %)*	133	*(61.3%)*	61	*(64.2%)*	72	*(59.0%)*	
*Asthenia (n; %)*	30	*(13.8%)*	9	*(9.5%)*	21	*(17.2%)*	
*Chest pain (n; %)*	22	*(10.1%)*	16	*(16.8%)*	6	*(4.9%)*	
*Cough (n; %)*	18	*(8.3%)*	7	*(7.4%)*	11	*(9.0%)*	
*Lack of data (n; %)*	66	*(30.4%)*	26	*(27.5%)*	40	*(32.8%)*	
**COVID infection**							
Infection date (range)			(01/10/2019 -	01/11/2022)	(15/02/2020 -	03/11/2022)	
*Wave 1 and 2 (i.e. pre-vaccination) (n; %)*	147	(67.7%)	63	(66.3%)	84	(68.8%)	NS
Wave > 2 (i.e. post vaccination) (n; %)	70	(32.3%)	32	(33.7%)	38	(31.2%)	NS
Hospitalization	128	(56.14%)	38	(40.0%)	90	(73.8%)	*P* < 0.001
*Duration (in days) (Mean; SD)*	*27.0*	*(± 23.6)*	*14.38*	(*±* 12.4)	*32.28*	(*±* 25.2)	*P* < 0.001
*Oxygen therapy (n; %)*	111	(86.7%)	26	(68.4%)	85	(94.44%)	*P* < 0.001
*Intensive care (n; %)*	71	(55.5%)	15	(39.5%)	56	(62.22%)	*P* < 0.05
*ARDS (n; %)*	58	(45.3%)	11	(29.0%)	47	(52.22%)	*P* < 0.05
Intubation (n; %)	48	(37.5%)	7	(18.4%)	41	(45.56%)	*P* < 0.01

Pre-existing chronic pulmonary disease was uncommon. Chronic obstructive pulmonary disease was present in 6.0% of patients, emphysema in 2.3%, fibrosis in 0.5%, and other pulmonary diseases in 4.6%. Chronic heart disease was present in 11.1% of patients and renal insufficiency in 3.2%.

At the time of V/Q SPECT/CT, dyspnea was the most frequent symptom (61.3%). Asthenia, chest pain, and cough were reported less frequently. Detail about symptom data were unavailable for 66 patients. The mean delay between acute COVID-19 infection and V/Q SPECT/CT was 187.15 ± 140.1 days (range 44–1261). Ventilation imaging was performed using 99mTc-Technegas in 55.7% of patients and 81mKr in 44.3%.

### Pulmonary and Mediastinal Lesions

**Fig. 2:**
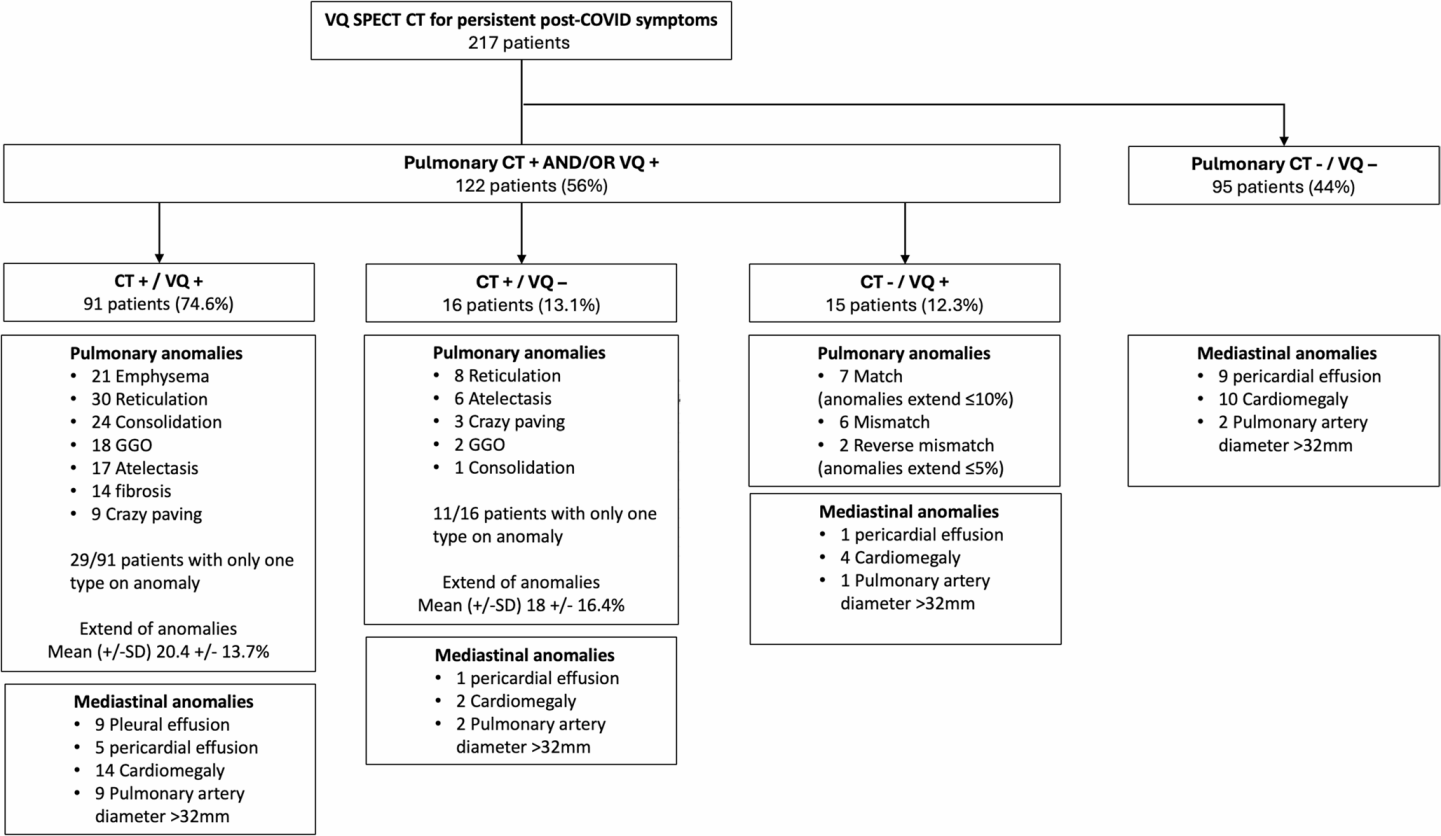
Flow chart

Of the 217 patients included in the registry, 122 (56.2%) presented abnormalities on CT and/or ventilation/perfusion imaging (Fig. 2). Among them, 91 (74.6%) exhibited combined CT and V/Q abnormalities, 16 (13.1%) had CT abnormalities only, and 15 (12.3%) showed functional abnormalities without CT abnormalities.

**Fig. 3:**
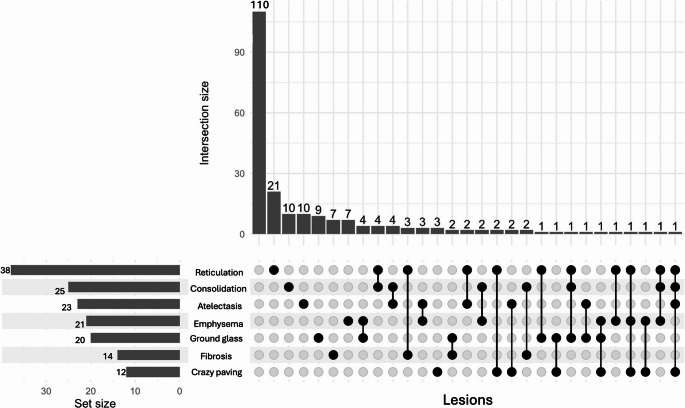
UpSet plot of the distribution and combination of pulmonary lesion observed in V/Q SPECT performed in a cohort of patient explore for persistent symptom after COVID infection. In the cohort, 110 patients presented with a normal V/Q SPECT/CT (column 1). Patients with a single lesion type are shown in columns 2–7 and 13. Combinations of two different lesion types are displayed in columns 8–12, 14–21, 23, and 25. Associations involving more than two lesion types were less frequent and are represented in columns 22, 24, 26, and 27

Among patients with parenchymal abnormalities (*n* = 107), reticulations were the most frequent lesion (38 patients, 35.5%), followed by consolidations (25 patients, 23.4%), atelectasis (23 patients, 21.5%), emphysema (21 patients,19.6%), ground-glass opacities (20 patients,18.7%), fibrosis (14 patients, 13.1%), and crazy-paving patterns (12 patients, 11.2%). A single parenchymal abnormality was present in 67 patients (62.6%), whereas 35 patients (32.7%) presented with two associated lesions, and 5 patients (4.7%) presented three or more different abnormalities. The distribution and the combination of these abnormalities are illustrated in Fig. 3.

Mediastinal abnormalities were frequently observed (*n* = 104). Among those patients, cardiomegaly was the most frequent abnormality (30 patients, 28.8%), followed by lymphadenopathy (27 patients, 26.0%), pericardial effusion (16 patients, 15.4%), pulmonary artery trunk enlargement ≥ 33 mm (14 patients, 13.5%), pleural effusion (9 patients, 8.7%).

Among patients with a normal pulmonary CT, cardiomegaly and pericardial effusion were each observed in 9 cases, suggesting that persistent respiratory symptoms may originate from extra-pulmonary mechanisms in a subset of patients.

### Functional Impact of Pulmonary abnormalities on Ventilation and Perfusion

**Fig. 4:**
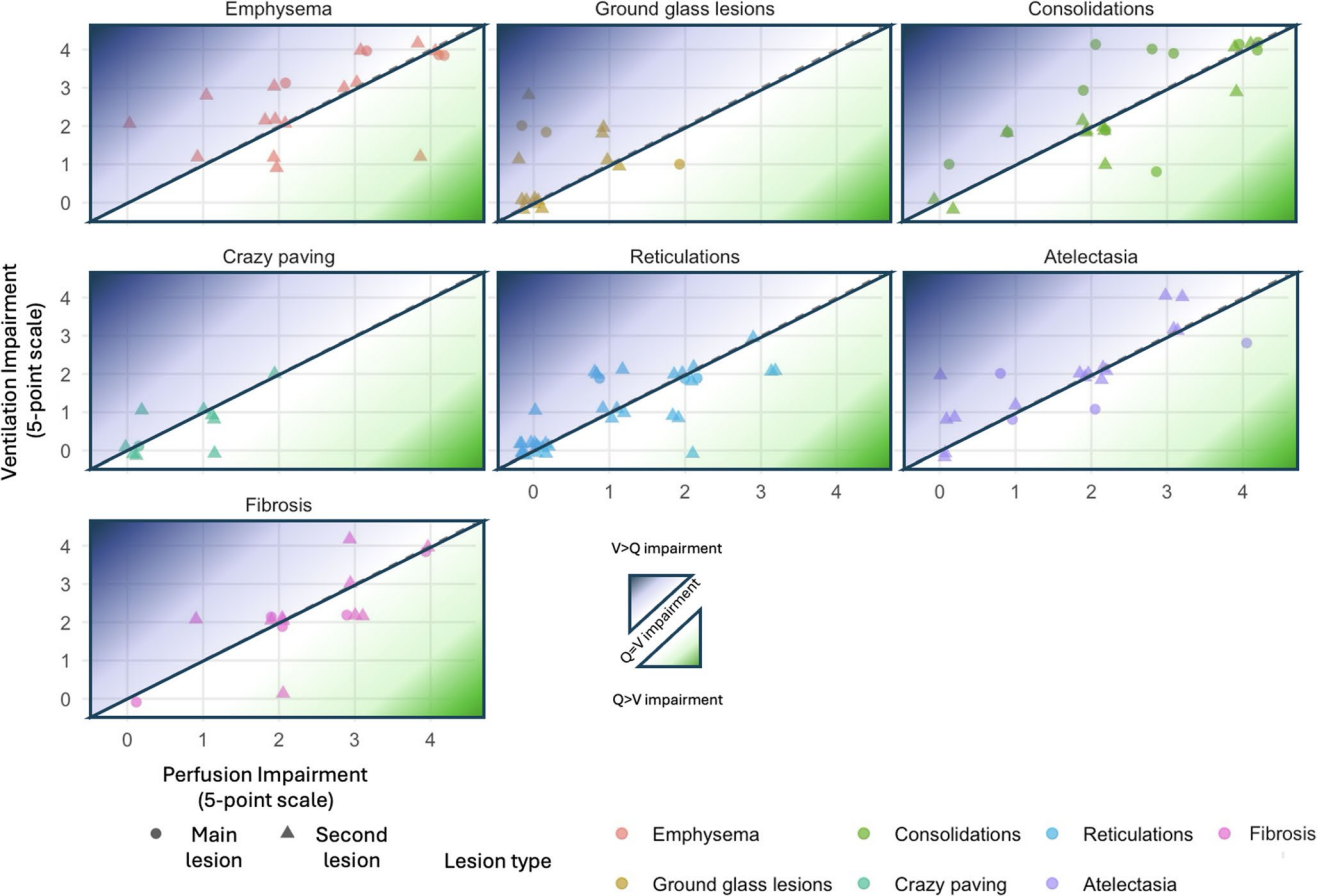
Quantitative repercussion of ventilation and perfusion alteration associated with lung lesions visualized in patients explored using V/Q SPECT after COVID-19 infection. Ventilation and perfusion were analyzed in all patients presenting parenchymal abnormalities involving at least 50% of one lung segment. When several lesion types were present in the same patient, the most extensive abnormality was analyzed. If a second abnormality involved a similar lung volume, it was evaluated separately. Ventilation and perfusion were independently scored using 5-point scale (0 = normal function to 4 = complete amputation of the function). Green area reflect V/Q mismatch (i.e. Q alteration superior to V alteration), blue area reflect reverse mismatch (i.e. V alteration superior than Q alterations), and black line correspond to match anomalies

**Fig. 5:**
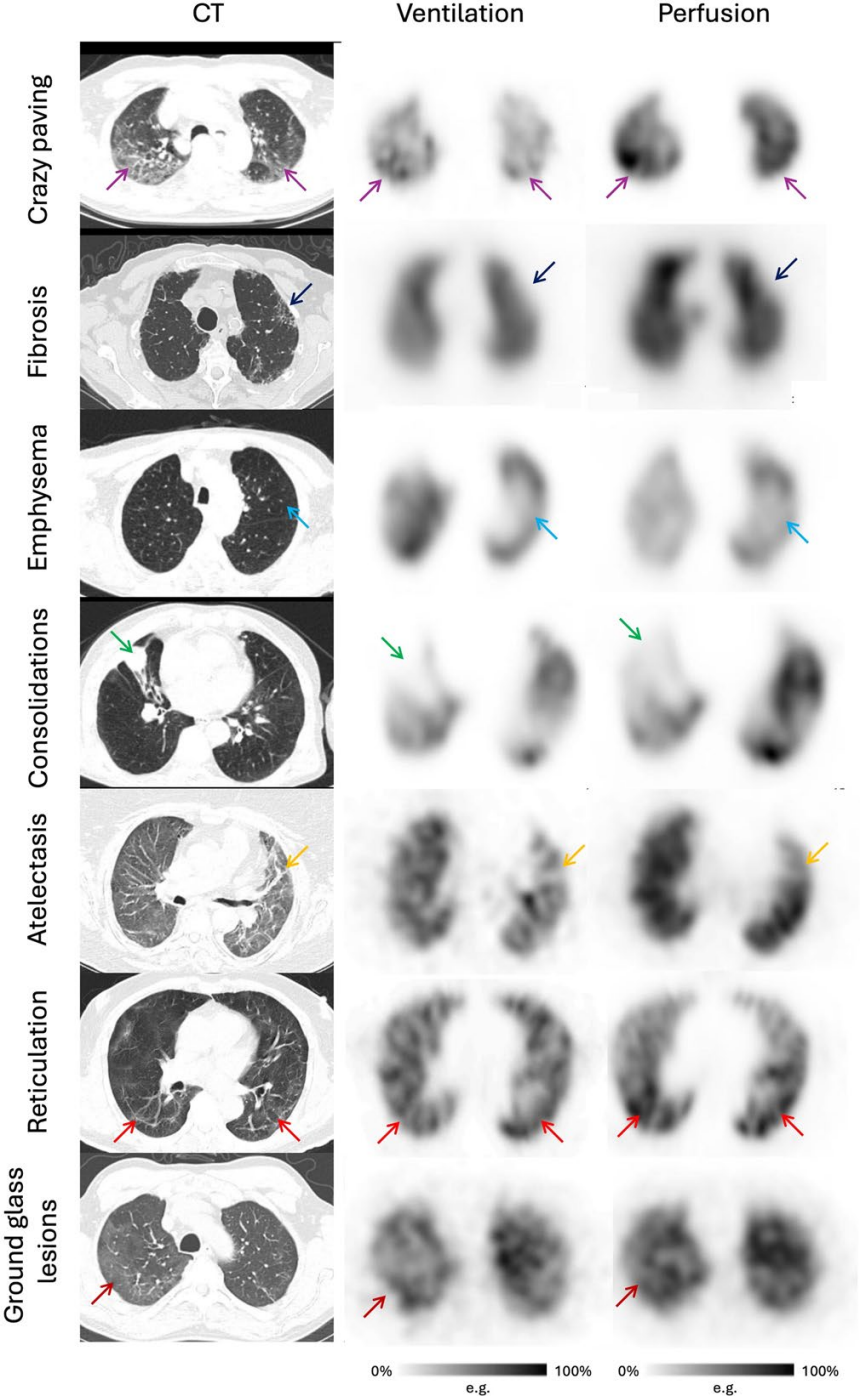
Representative examples of CT abnormalities and corresponding V/Q SPECT/CT findings in patients explored with V/Q SPECT after COVID-19 infection. Crazy paving was defined as ground-glass opacities associated with thickened interlobular and intralobular septa (purple arrows). This case illustrates mild perfusion impairment and preserved ventilation associated with the lesions in an 80-year-old patient without prior lung disease but with persistent symptoms more than two years after COVID-19 infection Pulmonary fibrosis encompassed reticulation with architectural distortion and honeycombing (navy blue arrows). The example shown lung lesions in a 78-year-old patient without prior lung disease, evaluated for ongoing respiratory symptoms after a COVID-19 infection requiring hospitalization and oxygen therapy during the acute phase. Severe perfusion impairment and moderate ventilation abnormalities were observed in relation to the CT lesions Emphysema corresponded to irregular enlargement of distal airspaces with destruction of alveolar walls (cyan arrows). This case involves a 69-year-old patient with known emphysema and symptoms suggestive of ongoing post-COVID-19 impairment. Moderate abnormalities were seen in both perfusion and ventilation, acquired here using krypton gas In the same patient, consolidation—defined as homogeneous opacification obscuring vascular markings (green arrows)—was also observed, associated with severe perfusion impairment and absence of ventilation in the affected region Atelectasis indicated regional loss of lung volume due to airspace collapse (yellow arrows). This example concerns a 71-year-old patient with dyspnea six months after a COVID-19 infection complicated by ARDS requiring ICU care and intubation. Mild perfusion impairment and moderate ventilation abnormalities were observed on SPECT in correspondence with the CT lesion Reticulation, defined as a mesh-like pattern of septal and intralobular lines (red arrows), showed no perfusion or ventilation abnormalities on SPECT in a 73-year-old patient without prior lung disease presenting with dyspnea and cough more than six months after COVID-19 infection requiring ICU care with oxygen but without intubation Ground-glass opacities were characterized by a hazy increase in attenuation preserving bronchovascular markings (burgundy arrows). The example shown is a 47-year-old patient with dyspnea seven months after a COVID-19 infection complicated by ARDS requiring ICU care. No perfusion defects were noted, whereas moderate ventilation impairment was observed on SPECT

The degree of ventilation and perfusion impairment varied according to the type of lesion (Fig. 4). Emphysema was associated with the most severe functional alterations, with mean perfusion and ventilation scores of 2.53 ± 1.17 and 2.68 ± 1.16, respectively (*n* = 19). Consolidations were similarly associated with high levels of functional impact (perfusion: 2.30 ± 1.33, ventilation: 2.48 ± 1.34; *n* = 23). Fibrotic lesions were associated with similar impairment (perfusion: 2.40 ± 1.06, ventilation: 2.20 ± 1.21; *n* = 15). Atelectasis were associated with mild impairment (perfusion: 1.65 ± 1.23, ventilation: 1.90 ± 1.12; *n* = 20). Crazy-paving patterns were associated with minimal alteration of perfusion and ventilation (both at 0.60 ± 0.70; *n* = 10). Reticulations, although the most frequent abnormality, were associated with limited functional consequences (perfusion: 0.97 ± 1.03, ventilation: 0.94 ± 0.95; *n* = 36). Ground-glass opacities were associated with the lowest perfusion impairment (0.38 ± 0.62), while ventilation was slightly more affected (0.94 ± 1.00; *n* = 16). Examples of the different morphological–functional patterns are illustrated in Fig. 5.

Among patients presenting CT abnormalities but no corresponding anomalies on ventilation or perfusion SPECT (*n* = 16; 13.1% of the overall cohort), the mean extent of abnormalities was 18% ± 16.4%. The CT findings were distributed as follows: 8 reticulations, 6 atelectasis, 3 crazy-paving patterns, 2 ground-glass opacities, and 1 consolidation. Notably, 11 of the 16 patients exhibited a single type of abnormality. In the subgroup of patients with ventilation and/or perfusion abnormalities but no corresponding CT findings (*n* = 15; 12.3% of the overall cohort), 7 cases showed matched defects (extent ≤ 10%), 6 showed mismatched perfusion defects, and 2 exhibited reverse mismatch patterns (extent ≤ 5%).

### Predictors of abnormal V/Q SPECT/CT

Subgroup analysis comparing patients with normal (*n* = 95) and abnormal (*n* = 122) V/Q SPECT/CT findings is summarized in Table 1. Age was a strong determinant of abnormal imaging. In the 18–35 year age group, patients with normal scans were significantly more frequent than those with abnormal scans (14.7% vs. 4.1% of their respective group, *p* < 0.01). This difference persisted in the 36–50 year group, where normal scans accounted for 32.6% compared with 14.8% of abnormal scans (*p* < 0.01). Conversely, patients older than 65 years were significantly overrepresented among abnormal scans, comprising 46.7% of abnormal scans versus only 13.7% of normal scans (*p* < 0.001).

Pre-existing chronic pulmonary disease was significantly associated with abnormal V/Q SPECT/CT with 18.1% of patients with pulmonary comorbidities presenting abnormal scans, compared with 5.3% with normal scans (*p* < 0.01). No significant difference was identified among patients without prior pulmonary disease (*p* = 0.32). There was a trend for patients with pre-existing chronic heart disease to exhibit abnormal rather than normal scans (14.8% vs. 6.3%, *p* = 0.053). No difference was observed in patients with renal failure or a history of pulmonary embolism. The COVID-19 wave during which infection occurred had no significant impact on imaging outcomes. From a technical point of view, patients with abnormal V/Q SPECT/CT findings were predominantly performed using ⁸¹ᵐKr ventilation (54.1% vs. 31.6%, *p* < 0.001). In univariate analysis (Table 2), age was identified as a significant predictor of abnormal imaging, (OR 1.045 per year 95% CI: 1.02–1.08; *p* < 0.01). Pre-existing chronic pulmonary disease was the strongest predictor (OR 5.113, 95% CI: 1.53–21.62; *p* < 0.05). Other comorbidities did not predict imaging abnormalities. Clinical presentation during long COVID was not associated with imaging outcomes, except for a history of less severe acute infection without oxygen therapy, which appeared protective (OR 0.2684, 95% CI: 0.1–0.69; *p* < 0,01).

**Table 2:** Univariate Analysis of Factors Predicting Abnormal V/Q lung SPECT/CT

	OR	[95% CI]	*P* value		VIF	R2 with other variables
**Patient characteristics**						
Age	1.045	[1.02–1.08]	0.0013	**	1.316	0.2403
Gender	1.227	[0.54–2.76]	0.6215	ns	1.307	0.2348
Chronic lung disease	5.113	[1.53–21.62]	0.0138	*	1.093	0.0848
Chronic heart disease	0.651	[0.19–2.38]	0.5014	ns	1.193	0.1615
Chronic renal disease	1.475	[0.17–33.45]	0.7530	ns	1.114	0.1021
Previous pulmonary embolism	2.310	[0.50–13.22]	0.3063	ns	1.072	0.0675
**Clinical presentation at the time of long COVID exploration**						
Dyspnea	1.270	[0.56–2.92]	0.5668	ns	1.186	0.1566
Asthenia	2.669	[0.92–8.77]	0.0844	ns	1.073	0.0678
Chest pain	0.411	[0.10–1.52]	0.1996	ns	1.089	0.0821
Cough	1.697	[0.43–7.37]	0.4610	ns	1.079	0.0732
**Clinical presentation at the time of COVID infection**						
No O2	0.268	[0.10–0.69]	0.0069	**	1.980	0.4950
No admission in reanimation	0.561	[0.21–1.52]	0.2507	ns	1.824	0.4519
PE during COVID infection	0.914	[0.37–2.25]	0.8448	ns	1.271	0.2134

## Discussion

This national multicenter study provides a comprehensive evaluation of pulmonary sequelae in long COVID using V/Q SPECT/CT and represents, to our knowledge, the largest cohort explored with this imaging modality to date. By combining morphological CT analysis with semi-quantitative ventilation and perfusion assessment, our results offer an integrated structural/functional perspective of post-COVID pulmonary abnormalities. The main findings demonstrate that certain post-COVID lesions remain markedly impaired several months after infection, whereas others, although morphologically visible, are associated with only mild functional consequences. These results refine the understanding of persistent respiratory symptoms in long COVID and highlight the added value of V/Q SPECT/CT for individualized patient evaluation.

Persistent pulmonary abnormalities after COVID-19 have been extensively reported, with impaired diffusing capacity emerging as the most frequent sequelae [6, 26]. Morphological changes are often attributed to a fibrosis-like restrictive pattern on high-resolution CT (HRCT) [27–29]. A meta-analysis reported a pooled prevalence of residual CT abnormalities of approximately 43% in post-COVID patients [26], which is closely consistent with the prevalence observed in our cohort despite the use of low-dose CT in our cohort. By contrast, the same meta-analysis reported a pooled prevalence of pulmonary fibrosis of 32% and of GGO of 34% substantially higher than the prevalence observed in our cohort (6%), where fibrosis was identified in only 14 patients and GGO in 12 patients. This difference is likely explained by the longer interval between infection and imaging in our cohort, as pulmonary fibrosis is considered a late-stage manifestation of lung injury [30]. Indeed, the mean delay between infection and imaging in our population was 187 days (range 44–126), suggesting that many early radiological changes had already evolved. Reticulations were particularly common in our series, which may represent intermediate or pre-fibrotic stages in the continuum of post-COVID lung recovery. Earlier cohorts imaged between 3 and 6 months after the acute infection reported higher prevalences of GGO, interlobular septal thickening, and reticulations [31–33]. Wu et al. observed persistent CT abnormalities in 78% of patients at 3 months, mainly GGO and reticulations, decreasing progressively at 6 and 9 months, although complete resolution was rare [28]. Luger et al. [34] reported incidence of GGO, reticulation and consolidation at 74%, 58% and 13% respectively at 2 month follow up to decrease to 44%, 43% and 1% at 1 year follow up. A recent follow-up study found that the proportion of COVID-19 survivors with residual lung abnormalities declined from 46% at 6 months to 36% at 3 years, with non-fibrotic lesions gradually resolving, while fibrotic-like lesions remained largely stable [35]. The Danish SECURe cohort [36, 37] provides a particularly relevant reference framework, as it also included V/Q SPECT at follow-up. In this cohort, ventilation–perfusion abnormalities were highly prevalent, at both early and intermediate follow-up appearing in 87% and 83% of patients respectively, with ventilation defects exceeding perfusion abnormalities. A similar pattern was observed in our cohort, in which most abnormalities affected both ventilation and perfusion, whereas isolated perfusion defects were uncommon. The SECURe cohort also reported that structural abnormalities on HRCT, particularly GGO and reticulations, often persisted up to 12 months despite partial improvement in pulmonary function.

A key strength of the present study lies in the semi-quantitative functional scoring applied to each lesion type, allowing direct comparison with our previously published cohort of patients explored during the acute phase of COVID-19 using the same methodology [25]. In the acute phase of the infection, the morphological abnormalities observed was predominantly consolidations, GGO, and crazy-paving patterns [38]. Consolidations and crazy-paving were associated with marked functional impairment (perfusion score 2.1 ± 1.0; ventilation score 3.0 ± 0.9), and (perfusion score 2.1 ± 1.1; ventilation score 2.4 ± 1.1), respectively. GGO also exerted both perfusion and ventilation at that stage (perfusion score 0.9 ± 0.6; ventilation score, 1.7 ± 1.0). In contrast, in the present long-COVID cohort, GGO and crazy paving patterns were associated with only minimal functional impairment (perfusion 0.38 ± 0.62; ventilation 0.94 ± 1.00) and (perfusion 0.60 ± 0.70; ventilation 0.60 ± 0.70), respectively. Although caution is warranted when comparing findings across different patient populations, this evolution is consistent with the natural resolution of acute inflammation, vascular congestion, and interstitial edema over time [39]. Conversely, emphysema, consolidations, and fibrosis (lesions more likely to reflect irreversible parenchymal damage) remained associated with substantial and persistent functional impairment. This clear dissociation between transient inflammatory lesions with spontaneous improvement and persistent structural abnormalities with lasting functional consequences supports the relevance of V/Q SPECT/CT imaging to evaluate the long-term respiratory consequences of COVID-19.

The combined morphological and functional information provided by V/Q SPECT/CT offers valuable insights into the heterogeneity of long COVID. The parallel impairment of ventilation and perfusion observed in most lesions suggests that persistent symptoms arise from combined airway and vascular mechanisms rather than from isolated microthrombotic or embolic processes. It is well established that COVID-19 is associated with a hypercoagulable state, leading to a substantial excess incidence of venous thromboembolism [40, 41] and pulmonary embolism during the acute phase of infection [42]. Delayed follow-up using dual-energy CT has demonstrated imaging features consistent with both acute and chronic pulmonary embolism, as well as two distinct patterns of perfusion abnormalities suggestive of persistent hypercoagulability and unresolved or sequelae microangiopathy [43]. V/Q SPECT/CT is widely recognized as a valuable imaging modality for the evaluation of pulmonary embolism in the acute setting [44] and during post-embolism follow-up [45]. Its clinical utility has also been demonstrated in patients with COVID-19 [46]. With regard to thromboembolic disease, pulmonary embolism was intentionally not detailed in the present analysis, as it was addressed in a dedicated publication derived from the same national registry [11]. In that study, 6 patients (2.8%) exhibited mismatched perfusion defects consistent with PE, including 3 previously undiagnosed cases. Post-embolic sequelae were uncommon (4.2%). These findings confirm that although thromboembolic disease remains a serious complication requiring prompt identification and treatment, it does not appear to be the predominant mechanism driving persistent symptoms in the majority of long COVID patients referred for V/Q scanning.

Beyond pulmonary parenchymal disease, our study also highlights the importance of extra-pulmonary thoracic findings. In patients with normal pulmonary CT, cardiomegaly was present in 10 cases, pericardial effusion in 9 cases, and pulmonary artery trunk enlargement ≥ 33 mm in 2 cases. These findings suggest that persistent dyspnea in long COVID may originate from extra-pulmonary mechanisms in a subset of patients, an aspect rarely addressed in most post-COVID imaging studies. V/Q SPECT/CT therefore offers a broader thoracic assessment than lung parenchymal analysis alone and may contribute to a more comprehensive etiological work-up of persistent respiratory symptoms. More generally, some abnormalities observed in our cohort are likely to represent long-lasting or permanent sequelae, particularly emphysema and fibrotic changes, which were associated with the highest degrees of functional impairment. In contrast, several findings typically considered transient, such as pleural or pericardial effusions and GGO, were associated with minimal functional impairment and may improve over time, potentially contributing to clinical recovery. This distinction between irreversible and reversible patterns is consistent with previous longitudinal CT studies reporting gradual resolution of inflammatory or edematous abnormalities, whereas structural remodeling tends to persist [28, 34, 47].

The optimal imaging strategy for long COVID remains to be clearly defined, as each imaging modality presents specific strengths and limitations [48]. Although widely available and useful at initial presentation, chest X-ray has limited sensitivity. Chest CT plays a central role in long-term assessment owing to its high spatial and contrast resolution, allowing detailed evaluation of lung parenchymal changes [31–33]. While MRI is not routinely used for lung imaging, it has shown promising results [49–51]. Although not suitable for routine clinical practice, ¹⁸F-FDG PET/CT may be useful in selected cases, particularly for detecting extra-pulmonary involvement and assessing both pulmonary and systemic sequelae during long-term follow-up [52, 53]. Finally, V/Q SPECT/CT may offer substantial added value by identifying small-vessel obstruction, characterizing functional parenchymal impairment, and monitoring COVID-19 survivors with persistent dyspnea. The identification of predictors of abnormal V/Q SPECT/CT further refines patient selection. Age and pre-existing chronic lung disease emerged as the strongest determinants of abnormal imaging, whereas a mild acute COVID-19 course without oxygen therapy was protective. These findings are consistent with previous studies showing that older age, severe acute infection, prolonged ICU stay, and mechanical ventilation are major risk factors for persistent radiological sequelae [29, 35, 54, 55]. From a clinical perspective, these data support a targeted use of V/Q SPECT/CT in patients with high-risk profiles (severe acute infections, prolonged ICU stay), or persistent unexplained dyspnea in older patients. Patients with such risk factors may benefit most from V/Q SPECT/CT imaging to clarify the mechanisms of their symptoms. A dedicated cost-effectiveness analysis could further define the optimal role of this modality in clinical pathways.

This study has several limitations. First, all scans were interpreted by a single expert, which may introduce observer bias and limit reproducibility. The morphological analysis was based on low-dose CT acquired with SPECT systems, which, although increasingly advanced, remain less precise than HRCT. This approach was chosen to ensure feasibility within a large multicenter cohort and to reflect real-world clinical practice. Moreover, despite the use of low-dose CT, the proportion of abnormal V/Q scans was similar to that observed in cohorts evaluated with HRCT. Second, the retrospective design may introduce recall bias and data from other imaging, respiratory test and follow-up were not available, preventing translational and longitudinal analysis of lesions. Third, V/Q SPECT/CT was performed based on clinical judgment rather than standardized clinical criteria, which may lead to overrepresentation of patients with more severe symptomatology. The low prevalence of pre-existing lung disease may reflect referral bias, as clinicians may have preferred alternative modalities for patients with known abnormalities. It also should be noted we observed a significantly higher use of 81 m-Krypton in the subgroup with abnormal V/Q SPECT. It is well established that this radioactive gas has different physical properties from the aerosol generated by Technegas. In particular, the gas may diffuse into emphysematous spaces in cases of pulmonary emphysema [56], whereas the aerosol could form tracheobronchial aggregates in situations of turbulent airflow, as commonly observed in asthma and COPD [57]. Nevertheless, both tracers are validated and exhibit comparable diagnostic performance [58] and they are routinely used in many nuclear medicine departments with similarly high reliability.

Despite these limitations, this study has several important strengths, including its nationwide multicenter design, centralized blinded review, and the largest population studied to date with V/Q SPECT/CT in long COVID. The nationwide registry enhances generalizability by capturing real-world clinical practices across diverse institutions. The combination of low-dose CT with functional lung imaging provides unique insights into the mechanisms underlying persistent respiratory symptoms and reinforces the complementary value of V/Q SPECT/CT in this heterogenous clinical setting.

## Conclusion

In conclusion, V/Q SPECT/CT emerges as a valuable and discriminating imaging modality for the evaluation of persistent respiratory symptoms in long COVID. By combining low-dose CT with ventilation and perfusion imaging, it provides an integrated assessment of structural abnormalities and their functional consequences, with clear added clinical value in a substantial proportion of patients. V/Q SPECT/CT may help to differentiate transient inflammatory lesions from persistent structural abnormalities associated with lasting functional impairment. Our results support a targeted use of V/Q SPECT/CT in selected patients with unexplained post-COVID dyspnea or high-risk profiles. Prospective studies including longitudinal follow-up and medico-economic evaluations are now warranted to better define its role in routine clinical practice.

## Data Availability

The datasets generated during and/or analysed during the current study are available from the corresponding author on reasonable request.

## Abbreviations

ARDS: Acute Respiratory Distress Syndrome
CT: computed tomography
CTPA: computed tomography pulmonary angiography
COPD: Chronic Obstructive Pulmonary Disease
ERS: European Respiratory Society
ESC: European Society of Cardiology
GGO: Ground Glass Opacities
HRCT: High Resolution computed tomography
ICU: Intensive Care Unit
OR: Odds ratios
PE: pulmonary embolism
SFMN: Société Française de Médecine Nucléaire (i.e. French Society Of Nuclear Medicine)
SPECT: single-photon emission computed tomography
V/Q: Ventilation/perfusion
